# Captive-reared migratory monarchs fly in the wrong direction: a critique of Wilcox *et al.*

**DOI:** 10.1093/conphys/coab063

**Published:** 2021-08-18

**Authors:** Andrew K Davis

**Affiliations:** Odum School of Ecology, University of Georgia, Athens, GA 30602, USA

Rearing monarch butterflies in captivity for release into the wild is an activity pursued by a growing number of citizens, despite calls from scientists and conservation groups about the risks to the overall population (Monarch Joint Venture, 2018; Pelton, 2018). One of the more well-publicized risks identified thus far is that monarchs raised indoors may be lacking a key developmental feature: reduced navigational sense when harnessed in a flight simulator (Tenger-Trolander *et al.*, 2019; Tenger-Trolander and Kronforst, 2020). This argues that some yet-to-be-identified environmental cue from the outdoors is needed for proper development of migration/navigational ability. Still, some indoor-reared monarchs do successfully reach their Mexico or California destination (Maeckle, 2018; James and Kappen, 2021), which raises the possibility that at least some metamorphosed adults can still regain their orientation sense once on their journey. Wilcox *et al.* (2021) recently tested this idea using a combination of flight simulator tests and releases of tracked monarchs from their location in southern Ontario. Their simulator tests confirmed that late summer-reared monarchs appear to show random orientation, consistent with prior experiments. They next released some of these monarchs after harnessing them with radio transmitters, which can track animal movement using a novel system of receiving towers called Motus (Taylor *et al.*, 2017). Their results showed that the monarchs travelled in a uniform direction (generously referred to as ‘southerly’ by the authors), and the authors claimed that this is evidence that reared monarchs can ‘regain’ their navigational ability once released. This claim is unjustified, as explained below.

It is true that the monarchs released by Wilcox *et al.* (2021) travelled in a more-or-less uniform direction (as opposed to random, as in the flight simulator), but importantly, the monarchs’ overall direction was *southeast*, which is not the direction leading to Mexico from southern Ontario (southwest). In fact, based on bearings of all released monarchs provided in their supplemental file, it is clear that not one monarch from their study showed the correct heading (below; see Fig. 1). This issue is the linchpin of the argument they are making—that reared monarchs have regained their ‘normal’ navigational ability. Of note here is that the authors did not have a control group for comparison that, ideally, would have been comprised of wild-caught migrants, which then would be harnessed with the same Motus tracking devices. If the trajectory of reared monarchs matched that of wild monarchs using this tracking system, then it might be possible to make this claim. Instead, the authors referred to a prior study (using the same tracking system) where a handful of wild monarchs showed a south-southeastern orientation when released at a location 250 km north of theirs (Knight *et al.*, 2019).

**Figure 1:**
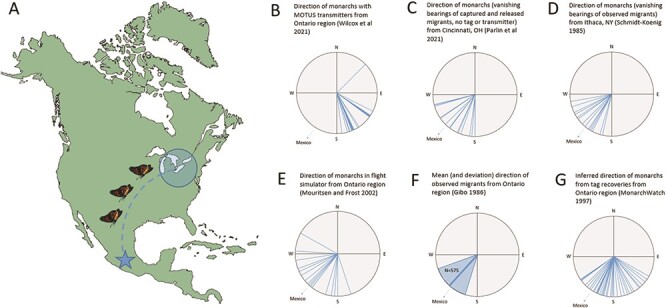
Comparison of reported flight trajectories of fall-migrating monarchs from the southern Ontario and surrounding region (A). Wilcox *et al.* (2021) tracked monarchs after outfitting with radio transmitters (B). Parlin *et al.* 2021 recorded vanishing bearings of captured and released migrants (C). Schmidt-Koenig (1985) observed directions of wild migrants in New York state (D). Mouritsen and Frost (2002) evaluated the navigation of wild migrants from southern Ontario with a flight simulator (E). Gibo (1986) observed migrating monarchs in southern Ontario and recorded flight directions of over 500 individuals (shown here is the mean direction and variance, as reported; F). Finally, monarch travel directions can be inferred from maps of points of origin and recovery of tagged individuals (MonarchWatch, 1997) (G). All compass images redrawn from material presented in cited articles. Except in (F), all lines represent the track of a single monarch. All lines are drawn at the same length, for simplicity.

The normal direction of travel of migrating monarchs from this region of North America is very clear, based on multiple prior navigational studies conducted from southern Ontario and the surrounding area in the past 40 years (Fig. 1). These include flight simulator tests (Mouritsen and Frost, 2002), vanishing bearings of released migrants (Parlin *et al.*, 2021), direct observations of wild migrants (Schmidt-Koenig, 1985; Gibo, 1986) and even inferred tracks from tagged-and-recovered monarchs (MonarchWatch, 1997); though tracks drawn from tagging data are not as strong as evidence from direct observations of flight. Taken together, these separate pieces of evidence show that ‘normal’ migration in this region is a southwestward flight, or at the very least, southward—not southeast, which is the direction most of the reared monarchs appeared to travel. The reason(s) for this skewed flight trajectory would need to be examined with further tests, perhaps beginning by assessing the functionality of reared monarchs’ innate internal compass (Nguyen *et al.*, 2021).

Regardless of the navigational issue, another limitation of the study by Wilcox *et al.* (2021) is the means of tracking the released monarchs; while this tracking system may work well for larger animals (birds, bats), the jury is still out as to whether it is appropriate for insects. The authors state, almost in passing, that the Motus transmitters weigh 200 mg. Monarchs in that region have an average weight of 525 mg (Brindza *et al.*, 2008). The authors stated that they selected monarchs that weighed over 300 mg. In essence then, these monarchs were likely carrying a device that weighed half their body weight. While it is clearly possible for monarchs to achieve flight with this extra weight, as evidenced by the fact that some were detected many kilometers away, it is a stretch to call this ‘normal’ flight, since they would be so burdened. In fact, in the prior tracking study cited to bolster their case, Knight *et al.* (2019) state ‘Most tagged monarchs could not sustain flight after release and were kept for one to two nights, fed honey–water solution and released in the morning.’ To be fair, it is not clear if this statement implies the monarchs were burdened, or if the tagging was too late in the day for release. Another (newly identified) problem with this transmitter approach is that even the glue used for attachment could affect monarch flight and/or health. Recently, Parlin *et al.* (2021) evaluated the survival of monarchs that had had rods glued to their cuticle (using similar glue as the study in question) and observed 100% mortality after 5 days. This evidence argues that the procedure for attaching the harness is stressful and/or that the glue itself imparts a significant cost even days after attachment. Thus, given the variety of problems associated with using this tracking system on insects, any conclusions drawn from it (including the study by Knight *et al.,* 2019) must be tempered. For full disclosure, flight mill tests in my own laboratory involve glueing rods to monarchs for attaching to the mill (Davis *et al.*, 2012), but these monarchs are never expected to fly on their own in the wild.

Even if this study’s conclusions were eventually borne out with further tests, the navigational issue is only one of many potential risks identified with captive rearing, such as the fact that reared monarchs show reduced physical strength and have paler wing colour (Davis *et al.*, 2020), which is a known indicator of poor flight propensity and/or migration success (Davis *et al.*, 2012; Hanley *et al.*, 2013) for monarchs. In fact, a recent re-examination of tag recovery records of reared and wild monarchs showed that migration travel distance of reared monarchs was only one quarter of that of wild individuals (Tenger-Trolander and Kronforst, 2020), which is consistent with reared monarchs having poor flight ability and/or reduced flight strength. Captive rearing also carries risks of spreading disease, which itself is a known detriment to migration success (Bradley and Altizer, 2005). Thus, for Wilcox *et al.* (2021) to suggest that their results support the idea that captive rearing is a viable conservation strategy to help the monarch migration is a gross overstatement. Moreover, it runs counter to the message being spread by monarch and insect conservation organizations (Monarch Joint Venture, 2018; Pelton, 2018) that argue that the risks of this practice are too great.

Regarding the study by Wilcox *et al.* (2021), I would argue that these results are preliminary at best and should have been paired with true controls (wild migrants) before submission of this study for publication, and certainly before making claims about the safety of captive rearing for the monarch population. Given the declines in the number of monarchs successfully reaching their winter destination (Agrawal and Inamine, 2018), efforts should be focused on minimizing human impacts on migration ability of the population, not on populating the migratory generation with poor fliers.

## References

[ref1] Agrawal AA, Inamine H (2018) Mechanisms behind the monarch’s decline. Science 360: 1294–1296. 29930122 10.1126/science.aat5066

[ref2] Bradley CA, Altizer S (2005) Parasites hinder monarch butterfly flight: implications for disease spread in migratory hosts. Ecol Lett 8: 290–300.

[ref3] Brindza L, Brower LP, Davis AK, Van, Hook (2008) Comparative success of monarch butterfly migration to overwintering sites in Mexico from inland and coastal sites in Virginia. J Lepid Soc 62: 189–200.

[ref4] Davis AK, Smith FM, Ballew AM (2020) A poor substitute for the real thing: captive-reared monarch butterflies are weaker, paler and have less elongated wings than wild migrants. Biol Lett 16: 5. 10.1098/rsbl.2019.0922 PMC7211457 32264783

[ref5] Davis AK, Chi J, Bradley CA, Altizer S (2012) The redder the better: wing color predicts flight performance in monarch butterflies. PLoS One 7: e41323. 10.1371/journal.pone.0041323. 22848463 PMC3405115

[ref6] Gibo DL (1986) Flight strategies of migrating monarch butterflies (*Danaus plexippus* L.) in southern Ontario. In W Danthanarayana, ed, Insect Flight. Springer, Berlin Heidelberg.

[ref7] Hanley D, Miller NG, Flockhart DT, Norris DR (2013) Forewing pigmentation predicts migration distance in wild-caught migratory monarch butterflies. Behav Ecol 24: 1108–1113.

[ref8] James DG, Kappen L (2021) Further insights on the migration biology of monarch butterflies, *Danaus plexippus* (Lepidoptera: Nymphalidae) from the Pacific Northwest. Insects 12: 25. 33672834 10.3390/insects12020161 PMC7917764

[ref9] Knight SM, Pitman GM, Flockhart DTT, Norris DR (2019) Radio-tracking reveals how wind and temperature influence the pace of daytime insect migration. Biol Lett 15: 5. 10.1098/rsbl.2019.0327 PMC6684972 31266418

[ref10] Maeckle M (2018) Five monarch butterflies tagged and released at San Antonio Festival made it to Mexico. In Texas Butterfly Ranch. https://texasbutterflyranch.com/2018/04/25/five-monarch-butterflies-tagged-and-released-at-san-antonio-festival-madeit-to-mexico/.

[ref11] Monarch Joint Venture (2018) Captive breeding and releasing monarchs. https://monarchjointventure.org/images/uploads/documents/Captive_Breeding_and_Releasing_Monarchs_oct2015.pdf.

[ref12] MonarchWatch (1997) Migration and tagging—summary of the Urquhart tag-recovery data, 1964–1994. https://www.monarchwatch.org/tagmig/urq1.htm.

[ref13] Mouritsen H, Frost BJ (2002) Virtual migration in tethered flying monarch butterflies reveals their orientation mechanisms. Proc Natl Acad Sci 99: 10162–10166. 12107283 10.1073/pnas.152137299 PMC126641

[ref14] Nguyen TAT, Beetz MJ, Merlin C, el, Jundi B (2021) Sun compass neurons are tuned to migratory orientation in monarch butterflies. Proc R Soc B Biol Sci 288: 9. 10.1098/rspb.2020.2988 PMC7935079 33622121

[ref15] Parlin AF, Stratton SM, Guerra PA (2021) Assaying lepidopteran flight directionality with non-invasive methods that permit repeated use and release after testing. Methods in Ecology and Evolution Online. 10.1111/2041-210X.13648.

[ref16] Pelton EM (2018) Keep monarchs wild: why captive rearing isn’t the way to help monarchs. Xerces Society Webpage.

[ref17] Schmidt-Koenig K (1985) Migration strategies of monarch butterflies. In MA Rankin, ed, Migration: Mechanisms and Adaptive Significance. Univ. Texas Contrib. Marine Sci., Austin, TX, pp. 786–798. 27 (Supplement).

[ref18] Taylor PD, Crewe TL, Mackenzie SA, Lepage D, Aubry Y, Crysler Z, Finney G, Francis CM, Guglielmo CG, Hamilton DJ et al. (2017) The Motus Wildlife Tracking System: a collaborative research network to enhance the understanding of wildlife movement. Avian Conserv Ecol 12: 11.

[ref19] Tenger-Trolander A, Kronforst MR (2020) Migration behaviour of commercial monarchs reared outdoors and wild-derived monarchs reared indoors. Proc R Soc B Biol Sci 287: 8. 10.1098/rspb.2020.1326 PMC7575518 32752991

[ref20] Tenger-Trolander A, Lu W, Noyes M, Kronforst MR (2019) Contemporary loss of migration in monarch butterflies. Proc Natl Acad Sci U S A. 116 (29): 14671–14676. 10.1073/pnas.1904690116. 31235586 PMC6642386

[ref21] Wilcox AAE, Newman AEM, Raine NE, Mitchell GW, Norris DR (2021) Captive-reared migratory monarch butterflies show natural orientation when released in the wild. Conserv Physiol 9: 1. 10.1093/conphys/coab032 PMC8355447 34386237

